# FASH: A web application for nucleotides sequence search

**DOI:** 10.1186/1751-0473-3-9

**Published:** 2008-05-27

**Authors:** Isana Veksler-Lublinksy, Danny Barash, Chai Avisar, Einav Troim, Paul Chew, Klara Kedem

**Affiliations:** 1 Department of Computer Science, Ben-Gurion University, 84105 Beer-Sheva, Israel; 2 Computer Science Department, 721 Rhodes Hall, Cornell University, Ithaca, NY 14853, USA

## Abstract

FASH (Fourier Alignment Sequence Heuristics) is a web application, based on the Fast Fourier Transform, for finding remote homologs within a long nucleic acid sequence. Given a query sequence and a long text-sequence (e.g, the human genome), FASH detects subsequences within the text that are remotely-similar to the query. FASH offers an alternative approach to Blast/Fasta for querying long RNA/DNA sequences. FASH differs from these other approaches in that it does not depend on the existence of *contiguous *seed-sequences in its initial detection phase. The FASH web server is user friendly and very easy to operate.

FASH can be accessed at

(secured website)

## Background

Recent discoveries [1] suggest that long RNA sequences, acting as natural sensors, exist in eukaryotic genomes and that such RNA sequences have not yet been found by commonly used bioinformatics methods. Although packages such as BLAST [2] and FASTA [3] are tremendously useful, alternative approaches may locate candidates that have been missed by traditional approaches.

## Algorithm

Our algorithm is based on the Fast Fourier Transform (FFT) and is similar to a method originally developed in the 80's [4,5]. We define a "Query vs. Text" matrix M where each entry (i, j) is assigned the value 1 if Query [i] is identical to Text [j], and 0 otherwise.

**Figure 1:**
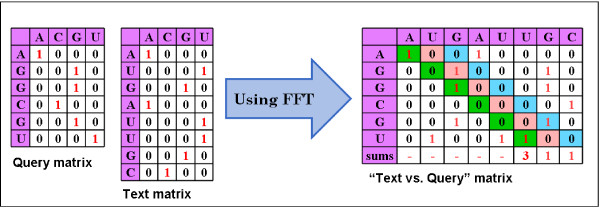
**Veksler-Lublinsky et al**. Matrices Q, T, and M.

This matrix is the product of matrices Q and T where Q is derived from the Query and T is derived from the Text (see Figure 1). Let *m *and *n *represent the length of the Query and the length of the Text, respectively. Matrix Q consists of *m *rows and 4 columns with one column for each base (e.g., *U*, *C*, *G*, and *A *for RNA). The entries in Q consist of 0s and 1s indicating which bases are present; there is exactly one 1 in each row. Matrix T is similar, but with *n *rows. It is easy to see that M, the "Query vs. Text" matrix, is equal to QT' where T' represents the transpose of T.

To locate a substring similar to the query string, we do not need the entire matrix M, we need just the sum along each diagonal of M. The sum of M's elements along diagonal *d *indicates the number of identities between nucleotides in Query [1..m] and in Text [d..d+m-1]. Using the FFT, we can efficiently calculate the sum for each diagonal of the matrix, obtaining the number of matches along each such diagonal in far less time than it takes to build matrix M.

Note that a gap (an unmatched nucleotide) has the effect of switching the "match-path" to an adjacent diagonal. If gaps are randomly distributed and if the Query sequence is sufficiently long then there should be enough similarity along one of the diagonals to detect the match. Thus, FASH works best for detecting remote homologs if the Query string is fairly long (say, 400 or longer).

Once we have located the significant diagonals, we apply traditional sequence alignment methods on the portions of the Text near significant diagonals. Significance is determined by straightforward statistical considerations. Assuming that each element of the diagonal is a single Bernoulli trial, the expected number of matches along a diagonal is *mp *where *m *is the length of the query, and *p *is the probability of a match in each position. The variance is *mp*(1 - *p*). For example, assuming each base has probability *p *= 0.25 of appearing in a particular position, if the Query is of length 600 then the expected sum along a random diagonal is (600)(0.25) = 150, with variance (600)(0.25)(0.75) = 112.5, and with a standard deviation *σ *≈ 10.61. In other words, the expected sum for a randomly chosen diagonal is 150 and a sum above 192.5 is more than 4*σ *from the mean. If four *σ*s are used for the significance threshold then false positives occur on less than 1 out of 10000 diagonals.

If *n *is very large (e.g., the size of a genome), we break the text sequence into pieces of size 2^13 ^where each such piece has an overlap of size 2^10 ^with the previous piece. We use the FFT to calculate sums for each piece separately.

## Complexity analysis

Using the FFT, all diagonal sums for M can be found in time *O*(*n *log *n*) where *n *is the length of the longer sequence (the Text, in our case). For our application, *n *is very large, so direct use of the FFT is impractical. Thus, we divide the Text sequence into pieces of size 2^*S *^with 2^*K *^overlap, where *S *and *K *are small constants (13 and 10 respectively, in our application). There are at most $\frac{n}{2^{S}-2^{K}}$ such pieces. For each piece, the FFT takes time 2^*s*^*log*2^*s*^, leading to an overall computation time of: $O(\frac{n}{2^{S}-2^{K}}\times 2^{S}log2^{S})=O(\frac{n}{2^{K}(2^{S-K}-1)}\times 2^{S}log2^{S})=O(n\times log2^{S}\times \frac{2^{S-K}}{2^{S-K}-1})=O(sn)$.

Additional time is needed for the dynamic-programming-based alignment methods that are run on the region around each significant diagonal, but this time is negligible compared to the FFT time.

## Server overview

FASH was designed as a user friendly application. The GUI is based on J2EE technology and was built using JSP pages and servlets. It runs on an Apache Tomcat web server. The application uses a Java N-Tier architecture containing the web layer, business layer, and data access layer. A MySQL database is used for saving all results from the *Request *and *Process *steps (see below), and for storing several pre-loaded genome files (taken from NCBI) that can be used as search Text.

We briefly describe some of FASH's features. For a more detailed description, we refer the reader to the documentation available at our web-site. The search is divided into three main steps.

**Figure 2:**
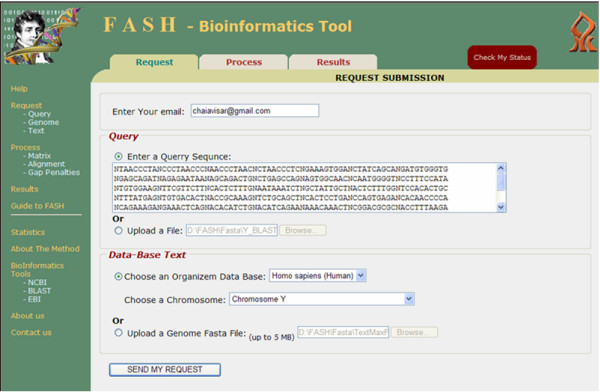
**Veksler-Lublinsky et al**. The request screen.

**Figure 3:**
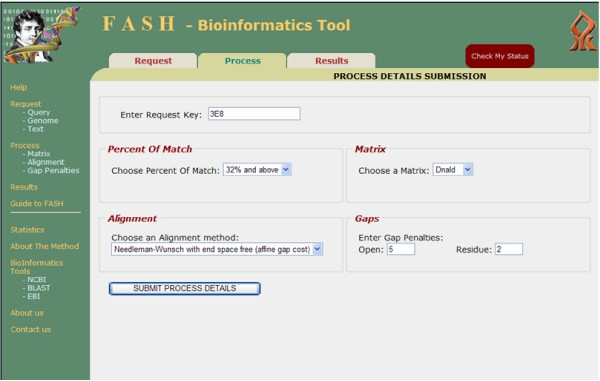
**Veksler-Lublinsky et al**. The process screen.

**Figure 4:**
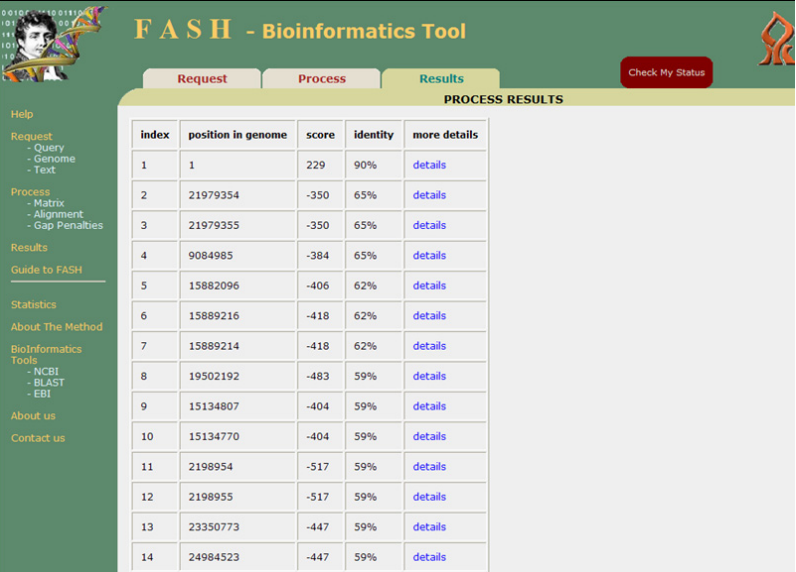
**Veksler-Lublinsky et al**. The results screen.

• Request: The user enters a Query and either chooses a pre-loaded Text genome or uploads a Text sequence (see Figure 2). After submitting the request, the user gets a serial key which will be needed for the *Process *step. All diagonal sums are calculated using the FFT. An email message is sent to the user when this step is complete.

• Process: The user enters a *Request *serial key and parameters: the alignment method, the scoring matrix, gap penalties, and the threshold (see Figure 3). After submitting parameters, the user gets a new serial key needed for the *Results *step. Sequence alignment methods are applied on all diagonals with sum above the specified threshold. For each *Request*, the user can submit several *Processes *each with its own serial key. The division into phases *Request *and *Process *enables the user to modify the parameters of the search, as well as the threshold, without reinvesting time in calculating sums using the FFT. An email message is sent to the user when the *Process *step is complete.

• Results: The user enters a *Process *serial key and indicates the number of alignment results to view (see Figure 4). FASH supports viewing up to 1000 results. Results are ranked by score and percent of identity between the Query and a position in the Text. The user can view the alignment, number and percent of identical positions, mismatches and gaps.

The user can check the *Request *or *Process *status at any time on the *Check Status *page. The answer is supplied from the online database.

The user can try the system with a guided example by following the "Guide to FASH" link. Information about different options and parameters is available via the "Help" link.

## Illustrative example where our application is advantageous

In order to illustrate a potential success of our method, we extracted a 560 nts sequence from chromosome Y of the human genome:

taaccctaaccctaaccctaaccctaaccctaaccctctgaaagtggacctatcagcaggatgtgggtgggagcagattagagaataaaagcagactgcct  gagccagcagtggcaacccaatggggtccctttccatactgtggaagcttcgttctttcactctttgcaataaatcttgctattgctcactctttgggtccaca  ctgcctttatgagctgtgacactcaccgcaaaggtctgcagcttcactcctgagccagtgagaccacaaccccaccagaaagaagaaactcagaacacatc tgaacatcagaagaaacaaactccggacgcgccacctttaagaactgtaacactcaccgcgaggttccgcgtcttcattcttgaagtcagtgagaccaaga  acccaccaattccagacacactaggaccctgagacaacccctagaagagcacctggttgataacccagttcccatctgggatttaggggacctggacagcc  cggaaaatgagctcctcatctctaacccagttcccctgtggggatttaggg

We searched this sequence both with the BLAST tool and with our FASH application, both were able to find the query. Next, we mutated every 7^*th *^nucleotide in the query sequence, and ran the program again. BLAST uses exact matching word (of size W) heuristic – it looks for short sequences of continuous matches longer than W and then extends them to produce an alignment.

In this run, we chose the minimal word size that BLAST enables (7 nucleotides). BLAST was not able to find our query in the human genome, while FASH ranked it as its highest hit, with 86% indentity to the query.

The mutated sequence was (mutated positions are underlined): taaccccaaccctgaccctacccctaaacctaactctctgacagtggatctatcaccaggatctgggtgcgagcagcttagaggataaaaccagactacct gagtcagcagcggcaactcaatggagtccctgtccatattgtggatgcttcggtctttctctctttccaataagtcttgcgattgctaactcttcgggtcctcactgct tttatgtgctgtgtcactcaacgcaaaagtctgctgcttcaatcctgacccagtgcgaccactaccccagcagaaacaagaaaatcagaaaacatctcaacatc tgaagaagcaaactacggacgtgccaccgttaagacctgtaatactcactgcgaggctccgcgacttcatccttgaactcagtgtgaccaataacccatcaattc aagacactctaggatcctgaggcaacccgtagaagtgcaccttgttgattacccagctcccatttgggatataggggccctggagagcccgtaaaatgcgctccta atctctgacccagatcccctttggggacttaggg

We observe above a case where our method is superior to BLAST. Admittedly, our test case is a synthetic example, but with the growing number and variety of biologically important problems it may well be that in the future our FASH application can be found helpful.

## Availability and requirements

Project Name: FASH

Project home page: .

Operating System(s): The FASH web application is platform independent.

Programming language: Java

Other requirements: None

License: None

Any restrictions to use by non-academics: None

## Abbreviations

FASH: Fourier Alignment Sequence Heuristics.

## Competing interests

The authors declare that they have no competing interests.

## Authors' contributions

All authors have read and approved the final manuscript. KK defined the problem and designed the project, IV supervised CA and AT code writing, IV, DB, CA, ET, PC, and KK tested and debugged the programs. All authors participated in the manuscript preparation.
